# Noninvasive and Multidisciplinary Approach to the Functional and Esthetic Rehabilitation of Amelogenesis Imperfecta: A Pediatric Case Report

**DOI:** 10.1155/2014/127175

**Published:** 2014-07-02

**Authors:** Juliana Feltrin de Souza, Camila Maria Bullio Fragelli, Marco Aurélio Benini Paschoal, Edson Alves Campos, Leonardo Fernandes Cunha, Estela Maris Losso, Rita de Cássia Loiola Cordeiro

**Affiliations:** ^1^Graduate Program in Dentistry, School of Dentistry, Positivo University, Rua Professor Pedro Viriato Parigot de Souza, 5300 Curitiba, PR, Brazil; ^2^Department of Pediatric Dentistry, Araraquara School of Dentistry, Universidade Estadual Paulista, UNESP, Araraquara, SP, Brazil; ^3^Master's Program in Dentistry, CEUMA University, UNICEUMA, São Luís, MA, Brazil; ^4^Department of Restorative Dentistry, Araraquara School of Dentistry, Universidade Estadual Paulista, UNESP, Araraquara, SP, Brazil

## Abstract

*Case Report*. An 8-year-old girl with amelogenesis imperfecta (AI) reported unsatisfactory aesthetics, difficulty in mastication, and dental hypersensitivity. The intraoral examination observed mixed dentition, malocclusion in anteroposterior relationships, anterior open bite, and dental asymmetry. A hypoplastic form of AI was diagnosed in the permanent dentition. A multidisciplinary planning was performed and divided into preventive, orthopedic, and rehabilitation stages. Initially, preventive treatment was implemented, with fluoride varnish applications, in order to protect the fragile enamel and reduce the dental sensitivity. In the second stage, the patient received an interceptive orthopedic treatment to improve cross-relationship of the arches during six months. Finally, the rehabilitation treatment was executed to establish the vertical dimension. In the posterior teeth, indirect composite resin crowns were performed with minimally invasive dental preparation. Direct composite resin restorations were used to improve the appearance of anterior teeth. *Follow-Up*. The follow-up was carried out after 3, 6, 12, and 18 months. After 18 months of follow-up, The restoration of integrity, oral hygiene, and patient satisfaction were observed . *Conclusion*. Successful reduction of the dental hypersensitivity and improvement of the aesthetic and functional aspects as well as quality of life were observed.

## 1. Introduction

Amelogenesis imperfecta (AI) is a disorder group of hereditary development that affects the dental enamel structure which is marked by clinical alterations without association with systemic abnormalities and diseases [1]. Apart of enamel defects, AI has been also associated with abnormalities in dental eruption, congenitally missing teeth, anterior open bite, pulpal calcifications, root and crown resorption, hypercementosis, root malformations, and taurodontism [2]. Furthermore, AI can affect partially or totally the teeth of both primary and permanent dentitions [2].

According to Aldred et al., [3], AI is a collective term for a number of conditions with defect of enamel formation. Many cases are inherited, either as an X-linked, autosomal dominant, or autosomal recessive trait. There are several classifications [3–6] based primarily on phenotype with the mode of inheritance being used in some systems as a secondary factor in allocating a case into a particular category [3]. According to the phenotypes and clinical aspects, AI can be classified into categories, such as type I that involves disturbances related to enamel secretion (hypoplastic), type II related to enamel maturation (hypomature), type III that affects the mineralization process (hypocalcified), and type IV, which is marked by the involvement of hypoplastic and hypomature enamel defects associated with taurodontism [7].

Clinically, AI is under dependence of disturbance types and presents with a thin enamel layer (hypoplastic), roughness texture (hypomature), a mottled appearance, opaque white to yellow-brown (hypocalcified), or association with one or two characteristics. To determine the presence of AI, an accurate diagnosis with other enamel defects and verification of alteration symmetric pattern linked to genetic inheritance are mandatory [3].

The main sequel to patients with AI is represented by dental sensitivity and breakdown of hard tissues due to weak mechanical properties of affected teeth [2]. Still, there are marked impacts on children and adolescents as a result of AI, including aesthetics, function, and psychosocial aspects [8]. Thus, attention should be taken to multiapproach treatment, aiming to determine the correct immediate and long-term planning follow-up [7].

Some cases of AI need orthodontic/orthopedic correction aiming at the improvement of function and aesthetic aspects, constituting an interdependent approach between a good alignment and restorative features [9]. In these situations, a preliminary wax-up planning is critical to the success of restorative procedure. The dental wax up will provide noninvasive tooth preparations guided by natural morphology with great benefit at the final clinical outcome [10].

Therefore, this paper aims to demonstrate, through a case report, a rehabilitation treatment by a noninvasive and multidisciplinary approach of a pediatric patient with hypoplastic form of AI.

## 2. Case Presentation

**Figure 1:**
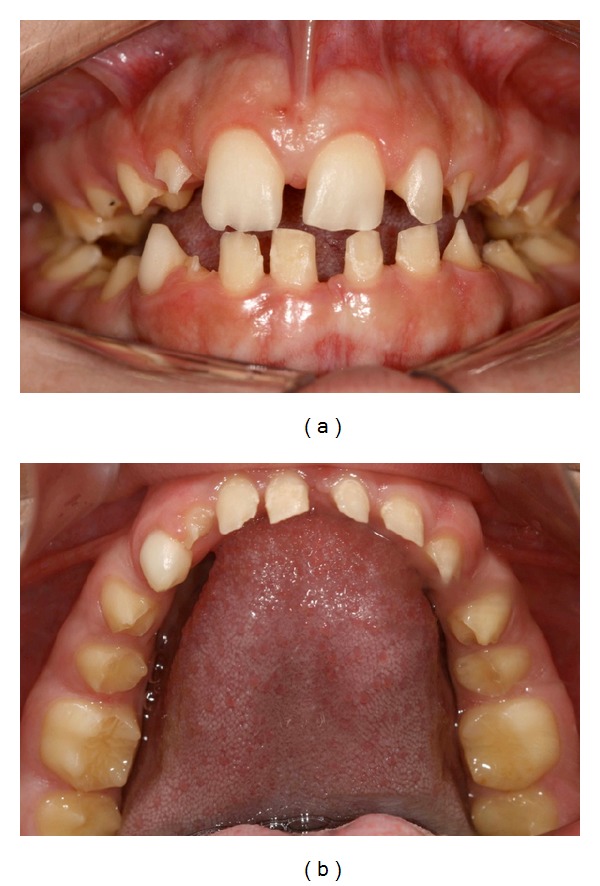
Initial intraoral aspect of 8-year-old patient with amelogenesis imperfecta.

An 8-year-old girl was referred to the Paediatric Dentistry Clinic, Araraquara Dental School (UNESP), for evaluation and treatment. Detailed medical and dental history was obtained from parent's speech. At intraoral examination, mixed dentition, congenitally missing of right upper lateral incisor (number 12), posterior cross-bite malocclusion, anterior open bite, dental asymmetry, and absence of caries activity were present. A thin enamel layer covering all teeth was verified, which resulted in painful sensitivity to mastication and decrease of aesthetics of anterior region stated by patient. Dental family history revealed that relatives suffered from similar dental conditions. The clinical and radiographic features were consistent with a possible diagnosis of hypoplastic AI (Figure 1).

The treatment planning was based on priorities related to dental sensitivity, masticatory function, cross-bite malocclusion, and aesthetics of patient. After discussion among professionals, parents, and patient, the treatment was planned in three different phases. The first phase was based on preventive approach with weekly application of 22,600 ppm of sodium fluoride (Duraphat, Colgate, Brazil) for four consecutive weeks to reduce dental sensitivity, to protect and prevent remaining structures from future loss, and, at the same time, to avoid caries development [11]. Reinforcement of oral hygiene was performed at every single appointment.

**Figure 2:**
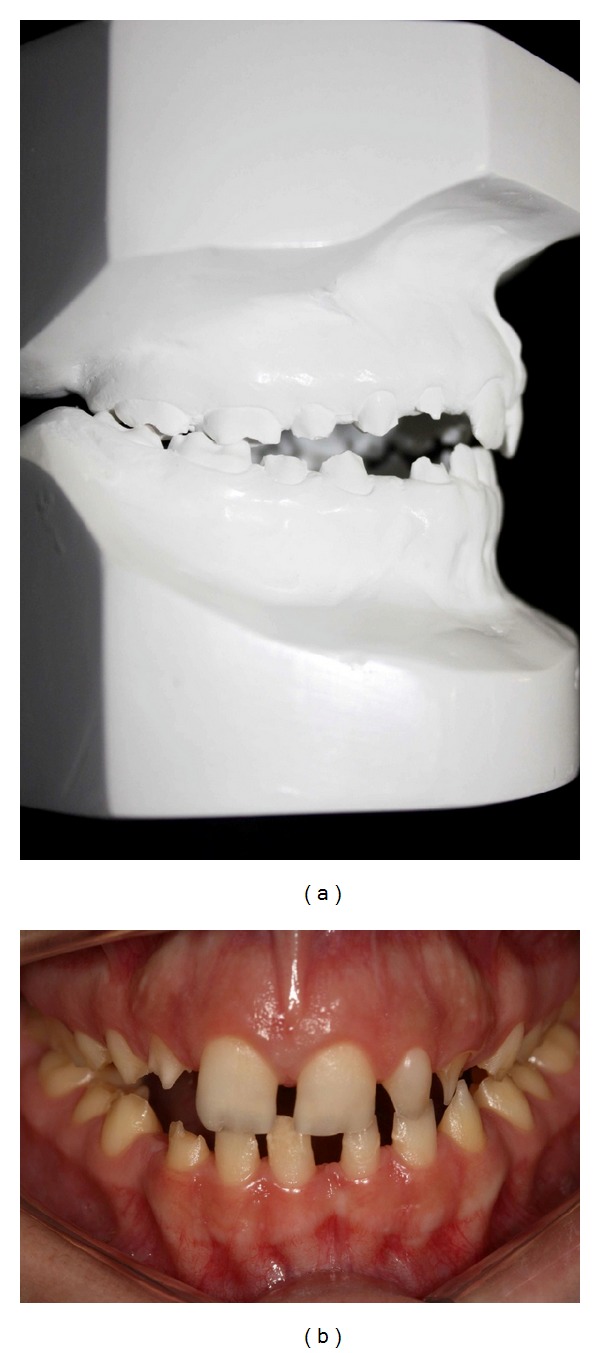
(a) The orthodontics appliance. (b) After orthodontic treatment (6 months).

The second phase consisted of orthopedic treatment to minimize posterior bilateral cross-bite and anterior open bite by the use of an intraoral interceptive device for 6 months (Figure 2). For orthopedic maxillary expansion, a removable appliance with screw was applied, used as palatal expander, that was activated once or twice a week [12]. The third and final phases started by the end of orthopedic treatment.

**Figure 3:**
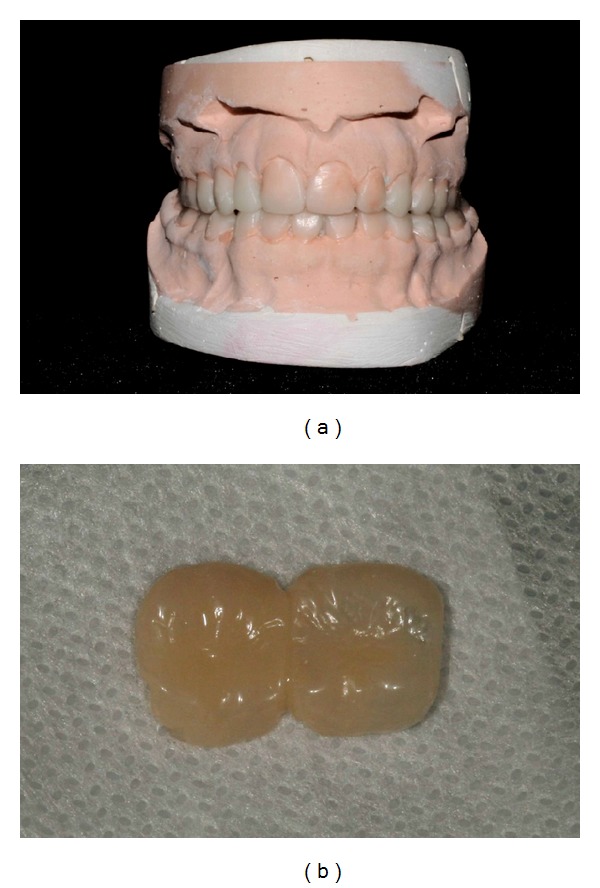
(a) Anterior diagnostic waxup. (b) Posterior resin crowns made according to the waxup.

A model cast of upper and lower teeth was done aiming to guide the position of posterior composite temporary crowns. After wax-up planning, models determined the need of 1 mm increase of occlusal vertical dimension (Figure 3(a)).

The upper and lower posterior teeth were minimally prepared to receive composite crowns. Tooth preparation involved only the smoothing of surface irregularities as well as the removal of unsupported enamel. After impressions of the preparations, composite resins crowns were obtained indirectly by VITA VM LC system (Vita Zahnfabrik, Germany) (Figure 3(b)), due to some clinical difficulties in children with AI, such as to maintain the mouth opening, to apply rubber dam, to control of the humidity, and more time to make the posterior tooth anatomy. The cement Rely X U200 was used (3M ESPE, St. Paul, MN, USA). The light polymerization was performed with a LED curing unit (radii-cal SDI, Bayswater, Victoria, Australia).

**Figure 4:**
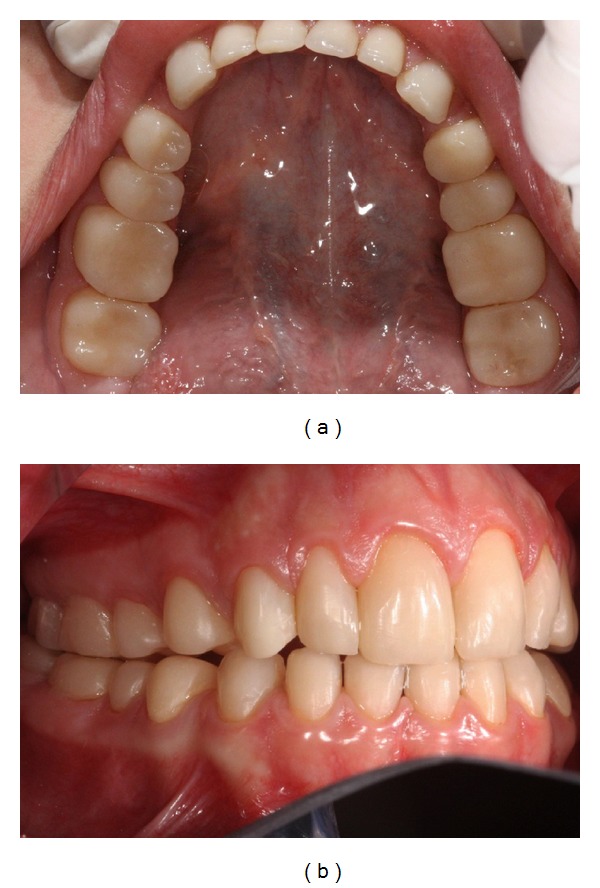
(a) Indirect and direct restorations in posterior and anterior lower teeth. (b) Final rehabilitation demonstrating aesthetic and functional result.

Subsequently, direct anterior restorations were performed with the support of a silicone guide (Express XT - 3M ESPE) directly, since it was easier to isolate and develop the anterior anatomy. An adhesive system was applied according to the manufacturer's instructions (Adper Scotchbond Multipurpose Plus, 3M ESPE, Seefeld, Germany). A thin layer of dentin shade (A2D) (Filtek Z350 XT, 3M ESPE, Seefeld, Germany) was first inserted to simulate the opacity of the dentin. The enamel shade was applied in the cervical third (A2E) and in the middle third. The enamel A1E was applied in the incisal third. Finishing and polishing procedures of the restorations were performed using sequential Sof-Lex discs (3M ESPE, Seefeld, Germany) (Figure 4).

**Figure 5:**
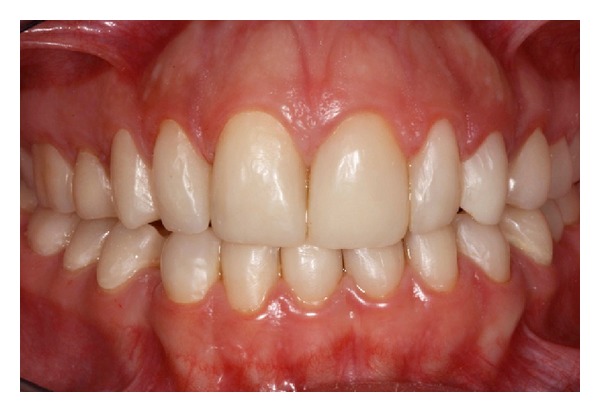
Follow-up after 18 months.

After the rehabilitation treatment, the patient and her relatives related satisfaction to the esthetics aspects as well as to the relief of the dental sensitivity, improving her masticatory function and quality of life. A follow-up after 18 months was performed to verify the condition of the treatment. The 6-month follow-up can be observed in Figure 5.

## 3. Discussion

The rehabilitation treatment is indicated in paediatric patients with AI. It is important to ensure the development of the craniofacial as well as psychology aspects of the children. The main goal of this treatment is to improve the quality of life, which includes improving the function, protecting the enamel structure, and reducing the sensitivity [13]. Additionally, the treatment proposed is to improve the aesthetic aspects.

Nowadays, there are several different materials and techniques available to restorative procedures, which have made it both exciting and confusing for dental practitioners. The treatment planning for AI is complex and varies according to the patient's age, symptoms, type and severity of the defect, and the intraoral situation at the time the treatment. Thus, a multidisciplinary approach, which includes pediatric, orthopedic, and operative dentistry, plays an important role in the final result of AI patients. For the ideal treatment, children affected by AI must be analyzed by individual case and in accordance with specialist from different expertise taking into account the limitations and the application of techniques [14], as demonstrated in the case presented.

Different treatment options for the treatment of AI-affected teeth have been proposed in the literature. A number of cases have given the predictability and high aesthetics achieved with complete crowns. However, this approach requires the removal of substantial amount of dental structure [14]. The treatment performed in this case was based on a multidisciplinary and conservative approach treatment, with minimal wear of the enamel structure with the support of the adhesive systems evolution and resin composite as well [2, 15, 16].

Patients affected by enamel hypoplasia present a thinner enamel, dental sensitivity, and more fragile structure, resulting in a more susceptibility to enamel breakdown and fractures during the masticatory function. To improve this condition, the first step for this case report was the preventive approach with 22,600 ppm of sodium fluoride. After four weeks, the patient related relief of the sensitivity. It was in accordance with data of the literature as stated by Petersson [11] that have been shown in a review study that the most fluoride preparations in combination with dentin fluid obstruction agents are beneficial to reduce the dental sensitivity. The main mechanism of fluoride to relieve dental sensitivity is its chemical ability to reduce and block fluid movements in the dentin tubules through formation of calcium-phosphorous precipitates as well as calcium fluoride (CaF_2_) and fluorapatite (FAp) [11].

Regarding the orthopedics treatment, the fragile structure affected by AI did not permit the placement of fixed orthodontic devices due to the shape and size of the affected teeth. Then, the orthopedics device aimed to expand the upper arch to treat the posterior cross-bite malocclusion; a retentive removable appliance with screw, used as palatal expanders, was applied that was activated once or twice a week [12]. This treatment improved the arches relationship in six months with a remarkable collaborative behavior of the patient.

The composite resins crowns for posterior teeth allowed a better relationship between the upper and lower arches, protected the enamel structure, and promoted an adequate vertical dimension for the patient, improving the masticatory function. This is an adequate treatment until the ideal age and development of the dental structure for a definitive rehabilitation. Cost-effective restorative techniques involving these composite-resin crowns were previously shown for the restoration of a young patient with amelogenesis imperfecta [17]. Another treatment option is the use of steel crowns, that is, an extremely durable restoration, being indicated for several clinical situations, such as large carious lesions involving multiple surfaces and children with high caries risk. However, present some disadvantages, such as its use in primary teeth and lack of esthetics [18].

The use of direct composite resin restorations to AI patient presented a favorable result as demonstrated at the 6-month follow-up. Papilla accommodation illustrates good interaction of the restorations with the soft tissue [9]; therefore, the biological aspects were successful. The direct restoration technique was also considered as an aesthetic approach for anterior teeth due to the mechanical properties and color stability [16]. Moreover, this technique allows the preservation of dental structure since the preparation is limited to affected areas with unsupported enamel [14, 19].

Based upon the complexity of this condition and considering the complications encountered during the treatment, the use of proper direct-bonded resin composite restorations associated with temporary composite crowns provided an excellent conservative provisional treatment for protection of teeth affected by AI and, at same time, the reestablishment of the quality of life and self-esteem of the patient.
